# Ambulatory electrocardiography, heart rate variability, and pharmacologic stress testing in cats with subclinical hypertrophic cardiomyopathy

**DOI:** 10.1038/s41598-022-05999-x

**Published:** 2022-02-04

**Authors:** Ashley L. Walker, Yu Ueda, Amanda E. Crofton, Samantha P. Harris, Joshua A. Stern

**Affiliations:** 1 Department of Medicine & Epidemiology, School of Veterinary Medicine, University of California-Davis, 2108 Tupper Hall, Davis, CA 95616-8732 USA; 2 Department of Clinical Sciences, College of Veterinary Medicine, North Carolina State University, Raleigh, NC 27607 USA; 3 Department of Cellular and Molecular Medicine, University of Arizona, Tucson, AZ 85724 USA

**Keywords:** Cardiology, Diseases, Medical research

## Abstract

The utility of ambulatory electrocardiography (AECG) to evaluate cats with subclinical hypertrophic cardiomyopathy (HCM) for arrhythmias and heart rate variability (HRV) is not well defined but may provide information regarding risk stratification. This prospective study used AECG to evaluate ectopy and HRV in subclinical HCM cats compared to healthy controls and is the first to implement a pharmacologic cardiac stress test. Twenty-three purpose-bred, Maine coon cross cats (16 HCM, 7 control) underwent 48-h of continuous AECG. Terbutaline (0.2–0.3 mg/kg) was administered orally at 24 and 36 h. Heart rate, ectopy frequency and complexity and HRV parameters, including standard deviation of normal R-R intervals (SDNN), were compared pre-terbutaline and post-terbutaline and across phenotype, genotype and sex. Genotype for an HCM-causative mutation was significantly associated with the frequency of supraventricular (P = 0.033) and ventricular (P = 0.026) ectopy across all cats. Seven HCM cats and zero healthy cats had a sinus arrhythmia. Mean heart rate was significantly higher post-terbutaline (p < 0.0001). HCM cats had significantly greater HRV compared to controls (SDNN: p = 0.0006). Male cats had significantly higher HRV (SDNN: p = 0.0001) and lower mean heart rates (p = 0.0001). HRV decreased post-terbutaline (SDNN: p = 0.0008) and changes in HRV observed between sexes were attenuated by terbutaline.

## Introduction

Hypertrophic cardiomyopathy (HCM) is the most common cardiac disease in cats, affecting approximately 15% of the general cat population^1–3^. HCM is characterized as thickening of the left ventricle myocardium in the absence of other hemodynamic or metabolic etiologies^4^. Feline HCM can be caused by an autosomal dominant missense mutation in the myosin binding protein C gene (MYBPC3) in the Maine Coon and Ragdoll breeds^5–7^. In people, mutations in more than eleven sarcomeric genes have been associated with HCM^8^. Feline HCM represents a highly comparable animal model for human HCM with similar pathophysiology, echocardiographic changes, and histologic findings. Potential outcomes of feline HCM are also similar to those in people, with some cats experiencing sudden cardiac death (SCD) as the first presenting complaint^9–11^. While cats that die suddenly are thought to have developed malignant arrhythmias, the antemortem clinical factors that increase the risk for SCD in subclinically affected cats remain incompletely understood.

In humans with HCM the development of non-sustained ventricular tachycardia is positively associated with the likelihood of experiencing SCD^12–14^. Ambulatory electrocardiogram (AECG) monitoring is commonly used in both human and veterinary medicine as a superior diagnostic tool for the identification of cardiac arrhythmias compared to standard in-hospital ECG^15,16^. A study of 178 human patients with HCM using AECG found a high prevalence of ventricular arrhythmias including ventricular premature complexes and non-sustained ventricular tachycardia^17^. Another human study using AECG and cardiovascular magnetic resonance imaging found that patients with greater myocardial fibrosis also had an increased frequency of ventricular tachyarrhythmias^18^. These patients had mild or asymptomatic HCM, suggesting that the development of myocardial fibrosis does not necessarily translate into clinical symptoms, however it does indicate a greater risk of SCD. While cats experiencing SCD have been identified to have a higher prevalence of interstitial fibrosis, subendocardial fibrosis and intramural arteriolosclerosis at necropsy, there have been no studies showing a correlation between fibrosis, ventricular arrhythmias and risk of SCD in cats^19^. Identifying cats with subclinical HCM that are arrhythmic, and particularly those with ventricular tachyarrhythmias on AECG, may be useful in guiding treatment, risk stratification, and preventing SCD.

Previous studies comparing the arrhythmia frequency and severity of cats with asymptomatic HCM to healthy cats using 24-h AECG monitoring have found differing results: one study found that cats with asymptomatic HCM had more frequent and complex ventricular and supraventricular arrhythmias, while the other failed to find a significant difference between groups, potentially due to differences in environmental stressors between the cats studied^20,21^. Another more recent study evaluated cats with compensated and decompensated HCM compared to healthy controls and found both HCM groups to have more ventricular arrhythmias but did not find a difference between HCM groups or a correlation to prognosis^22^. Ventricular arrhythmias in humans with HCM during exercise-induced cardiac stress are positively associated with a greater risk for SCD, possibly even more than the presence of ventricular arrhythmias when not undergoing stress testing^23^. Therefore, it is possible that cats with asymptomatic HCM that are at higher risk for SCD are more susceptible to the development of arrhythmias when faced with stressors and experiencing increased sympathetic drive. Ambulatory 24 or 48 h ECG monitoring and exercise stress testing are commonly performed in people with HCM to identify higher risk populations but have thus far not been established for use in cats with HCM in the clinical setting^24^.

In addition to the identification and quantification of arrhythmias, AECG monitoring can be used to evaluate heart rate variability (HRV). HRV is highly influenced by the autonomic nervous system, and therefore can be used as a measurement of sympathovagal balance and as a potential marker of cardiovascular disease^25–27^. In humans, a reduction in HRV has been shown to be a predictor of death in patients with progressive heart failure and a risk factor for development of left ventricular dilation in patients post-myocardial infarction^28,29^. Several studies evaluating HRV in people with HCM have found reductions in HRV parameters, with some differences depending on age or stage of disease^30–32^. For example, HRV was found to be more significantly reduced in patients that were symptomatic and in those pediatric patients that later experienced SCD. Spectral parameters of HRV have previously been evaluated in healthy cats using AECG to compare heart rate and HRV in the hospital versus in home setting, finding significantly higher heart rates in the hospital and changes to HRV indicating increased sympathetic tone^33^. HRV has not previously been assessed in cats with subclinical HCM and might be a useful tool for risk stratification for clinical outcome.

The aims of this study were firstly to use AECG monitoring to characterize the frequency and severity of arrhythmias as well as the HRV in a colony of Maine Coon-cross cats with subclinical HCM compared to healthy controls. Secondly, to create and implement a feline pharmacological cardiac stress test using terbutaline administration and assess its effects on heart rate and HRV as a means of validation in this population of cats.

## Materials and methods

### Animals

This study was approved by the Institutional Animal Care and Use Committee (IACUC) at the University of California, Davis. All methods were carried out in accordance with university guidelines, the approved IACUC committee protocol, and the ARRIVE guidelines and regulations. The animals used in the study were selected from a colony of Maine Coon-cross cats that were bred and raised at the UC Davis Feline HCM Research Laboratory, School of Veterinary Medicine. All cats were housed individually in an indoor enclosure throughout the study period. Prior to and following the study period cats were group housed. Cats were observed a minimum of twice daily and received water ad libitum and twice daily feedings. Diet was uniform across the colony without any supplements or medications beyond those in the study protocol. Cats underwent annual cardiovascular screenings including a physical examination, echocardiogram with simultaneous ECG, NTproBNP measurements and cardiac troponin-I measurements.

All cats were genotyped for the A31P mutation in the MYBPC3 gene, previously identified as causative for HCM in the Maine Coon breed^6^. Cats were chosen for the study if they had an echocardiogram performed within the previous ten months, their genotype for the A31P mutation was known, and their temperament would allow administration of oral medications. Genotypes were converted into mutant allele count for statistical analyses, with wild type = 0, heterozygous positive = 1, and homozygous positive = 2. The age, sex (intact male or female), weight, and most recent NTproBNP measurement for each included cat was recorded. No cats showing any clinical signs or evidence of being in congestive heart failure were included in the study.

### Echocardiography

Echocardiography was performed by a board-certified veterinary cardiologist (JS) with cats restrained in right and left lateral recumbency sequentially. Cats were sedated with intramuscular butorphanol (0.2 mg/kg) and acepromazine (0.05 mg/kg) at the time of echocardiography. Standard 2-dimensional, M-mode, and Doppler examinations were performed and recorded with analysis conducted using standard offline software. The maximal left ventricular wall thickness (septum or free wall) was recorded for each cat in both right parasternal long-axis 2D views and using 2D of M-mode, excluding insertion sites of moderator bands. Cats were classified as having an HCM phenotype if they met the following criteria prior to inclusion in the study: (1) left ventricular wall thickness of the interventricular septum, left ventricular free wall or both were ≥ 6 mm when measured from 2D or M-mode images or (2) left ventricular wall thickness of the interventricular septum, left ventricular free wall or both were ≥ 5.5 mm when measured from 2D or M-mode images and the cat had an NTproBNP measurement of > 99 pmol/L^34^. If cats did not meet either of these criteria for an HCM diagnosis, they were classified as healthy controls. The maximal left ventricular wall measurement (septum or free wall, whichever was greater) via M-mode from each cat’s most recent echocardiogram was recorded. Left ventricular outflow tract obstruction was noted to be present when turbulence was observed via color Doppler within the left ventricular outflow tract and the spectral Doppler velocity was > 1.9 m/s.

### Sedation and Holter placement

All cats were sedated with 0.25 mg/kg midazolam and 2.0 mg/kg of alfaxalone intramuscularly approximately 10 min prior to Holter device placement. The thorax of each cat was shaved, cleaned with 70% isopropyl alcohol, and dried with gauze. A commercially available, AECG device (H3 + Digital Holter Recorder, Mortara Instrument, Inc.) with three-channel recording was attached to each cat with three electrodes placed on the right thorax and two on the left thorax in a position to optimize ECG signal. Each monitor was initialized for 48 h of continuous recording. Monitors and leads were secured to each subject using skin-safe elastic adhesive tape and bandaging tape. Following placement, each subject was monitored until alert and able to stand at which time they were returned to the colony and placed in a separate enclosure from other individuals for the entire 48 h of AECG recording. After 48 h, cats were sedated with the same initial protocol for device removal, monitored for a minimum of 30 min and until determined to be fully recovered and then returned to their group housing enclosure.

### Terbutaline administration

After approximately 24 h of continuous AECG recording each cat was administered a dose of oral terbutaline sulfate. As an orally administered, beta agonist that is used clinically in cats, terbutaline sulfate was chosen as the best option. Based on the authors’ clinical experience, terbutaline would result in cardiovascular stimulatory effects with minimal invasiveness when compared to intravenous pharmacologic stress testing options used in humans such as dobutamine. The dose for each cat was between 0.2 mg/kg and 0.3 mg/kg, with cats weighing less than 4.0 kg receiving 0.625 mg and cats weighing more than 4.0 kg receiving 1.25 mg. After 36 h of continuous AECG each cat received a second, matched dose of oral terbutaline sulfate.

### Holter data acquisition and processing

All data from the AECG monitors was uploaded to the software analysis system, Vision 5 software (Mortara Instrument, Inc.) following device removal from each subject. The uploaded data was randomized via a standard random number generation system to allow for blinded analysis of the uploaded recordings. The authors that performed analysis of the uploaded recordings were therefore blinded to animal identification, previous echocardiographic findings, and genotype status. Analysis and interpretation of the uploaded AECG recording was made prospectively, in a similar manner to previous publication^35^. The software analysis system automatically annotates normal and abnormal complexes, however incorrect labeling of beat type and QRS timing occurs frequently in feline recordings. Therefore, all recordings were visually inspected on a beat-by-beat basis in their entirety and all mis-labeled beats were corrected in order to accurately determine the frequency and complexity of ectopy. QRS complexes labeled as normal but with incorrect timing, for example over the T or P wave, were manually corrected to allow for accurate HRV analysis. Portions of the recordings with motion-related artifact that was significant enough to preclude accurate labeling and interpretation was labeled as artifact, discarded and not quantified for analysis.

Supraventricular and ventricular arrhythmias were classified based on a complexity scale: 0 = no arrhythmias present, 1 = only single premature complexes, 2 = couplets, 3 = triplets, or 4 ≥ 4 consecutive ectopic beats. Ventricular arrhythmias were classified based on the instantaneous heart rate (HR) as premature (HR ≥ 160), accelerated idioventricular (HR = 100–159), or escape (HR ≤ 99 bpm) complexes. A sinus arrhythmia was noted to be present when the rhythm alternated between slower and faster heart rates in a cyclical pattern and all complexes in this pattern were sinus in origin. HRV was analyzed using standard time-domain techniques in accordance with published recommendations^36,37^. All of the time-domain measures of HRV were calculated for each one-hour period and averaged over the full disclosure using the Vision 5 ECG analysis software. The normal-to-normal (NN) intervals were calculated from one R wave to the next R wave for all sinus beats by the software. Based on the NN intervals, the mean, minimal, and maximal HR were recorded. The standard deviation of all NN intervals (SDNN), the standard deviation of the average NN intervals over 5 min (SDANN), the square root of the mean squared differences of successive NN interval (RMSSD), and the number of interval differences of successive NN intervals > 50 ms divided by the total number of NN intervals (pNN50) were obtained for each disclosure. The NN intervals were also converted to the triangular index value, defined as the integral of the density distribution of the NN intervals divided by the maximal density distribution.

### Statistical analysis

Data was tested for normality using the Shapiro–Wilk test. Normally distributed data are reported as mean and standard deviation; non-parametric data are reported as median and interquartile range. Parametric and non-parametric baseline population data were compared using an unpaired t-test and Mann–Whitney *U* test, respectively. Comparisons of ectopy frequency before and after terbutaline were made using a paired t-test for parametric data and the Wilcoxon matched pairs signed rank test for non-parametric data. Categorical variables were compared using a Fisher’s exact test. Direct comparisons of HRV for the HCM status, LVOTO, sex, and A31P genotypes over the observation period were performed using the Mann–Whitney *U* test or Kruskal Wallis test. Analysis of repeated measurements before and after terbutaline administration was performed using a linear mixed model to assess the effect of HCM status, LVOTO, sex, and A31P genotype as fixed effects. Simple and multiple linear regression via the least-squares model were used to assess for predictors of the total number of supraventricular and ventricular ectopic beats. Statistical significance was considered to be a p-value < 0.05.

## Results

### Characteristics of cats

A total of 23 cats were enrolled in the study and underwent 48 h of continuous AECG monitoring. Sixteen cats met the established criteria for a diagnosis of HCM based on echocardiogram and NTproBNP measurements, while seven cats were classified as healthy controls. A balanced distribution of genotypes for the A31P MYBPC3 mutation was achieved: 8/23 cats had 0 mutant alleles (wild type), 7/23 cats had one copy of the mutation (heterozygous), and 8/23 cats had two mutated alleles (homozygous). Baseline characteristics for the study population are displayed in Table 1. Of these, mutant allele count (p = 0.013), NTproBNP measurement (p < 0.0005), and maximal left ventricular wall thickness (p = 0.001) were significantly different between cats in the HCM phenotype group and healthy controls. All cats had a 48-h AECG monitor placed, however due to artifact, the length of recording analyzed varied. The median time of ECG recording analyzed was 39.85 h (IQR = 37.23–43.58). Seven cats were administered a dose of 0.625 mg oral terbutaline and 16 cats were given an oral dose of 1.25 mg at each dosing time per the study protocol, with all cats receiving a dose between 0.2–0.3 mg/kg. The first dose was administered 21–23 h into the analyzed recording and the second dose was administered 33–35 h into the recording time.

**Table 1:** Study population characteristics including signalment, genotype for the A31P MYBPC3 mutation, NTproBNP measurement, maximal left ventricular wall thickness, and presence of left ventricular outflow tract obstruction on echocardiogram.

Population characteristic	All cats (n = 23)	Control (n = 7)	HCM (n = 16)	p-value
Age (years)	3.64 ± 1.32	4.13 ± 1.51	3.43 ± 1.22	0.25
Sex (% male)	14/23 (60.9%)	3/7 (42.9%)	11/16 (68.8%)	0.36
Body weight (kg)	5.07 (3.10–5.68)	4.15 (3.05–5.20)	5.25 (3.53–6.57)	0.19
**MYBPC3 A31P allele count**	1 (0–2)	0 (0–1)	1.5 (1–2)	0.0131
0 or wild type (%)	8/23 (34.8%)	5/7 (71.4%)	3/16 (18.8%)	
1 or heterozygous (%)	7/23 (30.4%)	2/7 (28.6%)	5/16 (31.3%)	
2 or homozygous (%)	8/23 (34.8%)	0/8 (0.0%)	8/16 (50.0%)	
NTproBNP level (pmol/L)	52.0 (28.0–111.0)	26.0 (24.0–34.0)	63.0 (46.8–150.0)	0.0004
Maximal LV wall thickness (mm)	5.77 ± 0.98	4.86 ± 0.69	6.17 ± 0.82	0.0013
LVOTO present (%)	7/23 (30.4%)	0/7 (0.0%)	7/16 (43.8%)	0.057

Data are displayed as mean ± SD or median (IQR). Comparison is made between groups with p-values displayed.

### Heart rate, rhythm, and heart rate variability

**Figure 1:**
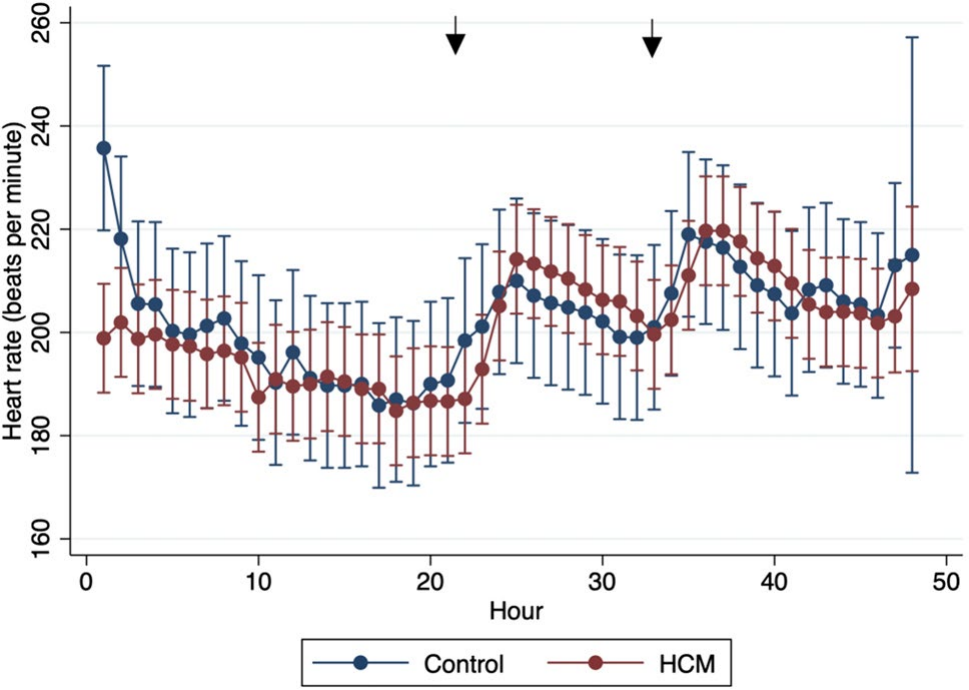
Mean heart rate (HR) plotted by hour for the entire AECG recordings separated by phenotype (subclinical HCM vs. control group). Arrows indicate when terbutaline was administered.

The median number of analyzed QRS complexes for each cat was 490,858 (IQR = 447,345–526,074). The mean heart rate over the entirety of ECG recordings was 201.7 ± 18.6 bpm (Table 2). When comparing the mean heart rates of cats in the resting state (prior to terbutaline administration) and stressed state (post-terbutaline administration), there was a significant difference (195.0 ± 22.56 bpm versus 209.2 ± 16.1 bpm, p < 0.0001) with higher mean heart rates in the stressed state. Analysis of the linear mixed model also reported significant differences with higher mean heart rate after terbutaline administration (p < 0.0001). There was no significant difference in the mean heart rate for cats in the HCM group versus the control group (p = 0.42). Figure 1 shows mean heart rate over the entirety of the recordings, demonstrating similar rates between the two phenotypic groups and the rise in heart rate for both groups in the stressed state.

**Table 2:** Holter recording data for the 23 cats for both the entire recording and the portion of recordings before and after terbutaline administration.

Holter parameter	Overall	Resting	Stressed	p-value (resting vs. stressed)
Heart rate	201.7 ± 18.6	195.0 ± 22.56	209.2 ± 16.1	< 0.0001
SV ectopic beats	2 (0–7)	1 (0–5)	0 (0–2)	0.181
**SV ectopy complexity**	1 (0–1)	1 (0–1)	0 (0–1)	0.0781
No ectopy	n = 6	n = 7	n = 15	–
Level 1	n = 13	n = 13	n = 7	–
Level 2	n = 3	n = 3	n = 0	–
Level 3	n = 0	n = 0	n = 0	–
Level 4	n = 1	n = 0	n = 1	–
V ectopic beats	8 (2–38)	5 (2–19)	3 (0–13)	0.0587
**V ectopy complexity**	1 (1–4)	1 (1–1)	1 (0–2)	0.7351
No ectopy	n = 1	n = 4	n = 7	–
Level 1	n = 14	n = 14	n = 10	–
Level 2	n = 1	n = 3	n = 2	–
Level 3	n = 0	n = 0	n = 0	–
Level 4	n = 6	n = 2	n = 4	–

Data are displayed as mean ± SD if normally distributed or median (IQR) if non-parametric.

**Figure 2:**
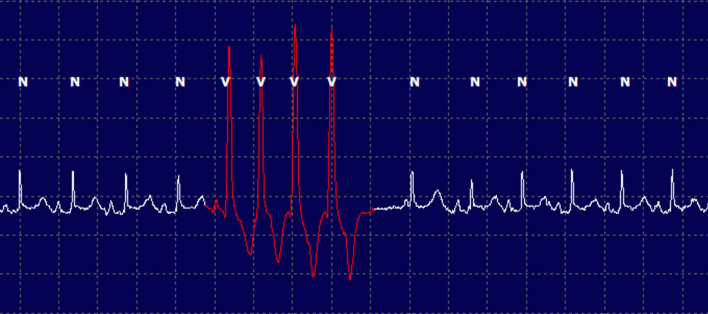
Example of ventricular tachycardia (complexity level 4) obtained via AECG in one cat from the HCM group.

The median number of supraventricular ectopic beats over the entire recording period was 2 (IQR = 0–7) with a median complexity level of 1 (IQR = 0–1). Seventeen of the 23 cats (73.9%) had at least one supraventricular premature complex over the recording period, the majority of which were single isolated complexes. The median number of ventricular ectopic beats over the recording period was 8 beats (IQR = 2–38) with a median complexity level of 1 (IQR = 1–4). Twenty-one of the 23 cats (91.3%) had at least one ventricular premature complex on their ECG recording. Most cats (14/23) only experienced single ventricular premature complexes, however there were six cats (26%) that had ventricular tachycardia (four or more complexes in a row) (Fig. 2). Two of these cats were in the control group and four were in the HCM group. The frequency and complexity of ventricular and supraventricular ectopic beats between the two groups are shown in Table 3.

**Table 3:** Ectopy frequency and complexity for the 23 cats for the entire recordings based on group.

Holter parameter	HCM (n = 16)	Control (n = 7)
Heart rate	200.4 ± 19.8	204.7 ± 16.8
SV ectopic beats	1.5 (0–200)	2 (0–23)
**SV ectopy complexity**	1 (0–4)	1 (0–2)
No ectopy	n = 5	n = 1
Level 1	n = 9	n = 4
Level 2	n = 1	n = 2
Level 3	n = 0	n = 0
Level 4	n = 1	n = 0
V ectopic beats	10 (1–202)	6 (0–151)
**V ectopy complexity**	1 (1–4)	1 (0–4)
No ectopy	n = 0	n = 1
Level 1	n = 11	n = 4
Level 2	n = 1	n = 0
Level 3	n = 0	n = 0
Level 4	n = 4	n = 2

Data are displayed as median (minimum–maximum) and the number of cats with each complexity level.

Simple linear regression was performed for both the total number of supraventricular and ventricular ectopic beats, assessing phenotype, sex, age, mutant allele count, NTproBNP, maximal left ventricular wall thickness, and the presence or absence of left ventricular outflow tract obstruction as possible predictors. Of these variables, none were significantly correlated with the total number of supraventricular ectopic beats. Only mutant allele count (genotype) was significantly correlated with the number of ventricular ectopic beats (p = 0.049; R^2^ = 0.17). Using multiple linear regression to evaluate the same variables as predictors of ectopy frequency, mutant allele count was significantly correlated with the total number of both ventricular (p = 0.026; R^2^ = 0.66) and supraventricular (p = 0.033; R^2^ = 0.66) ectopic beats (Table 4). When comparing the number and complexity of both supraventricular and ventricular ectopic beats before and after terbutaline administration there were no significant differences. Seven cats (30.4%) were noted to have a sinus arrhythmia at some point during the AECG recording. Of these, all seven cats were within the HCM phenotype group with zero cats in the control group having a sinus arrhythmia present on their recording (p = 0.06).

**Table 4:** Multiple linear regression evaluating for predictors of total ventricular and supraventricular ectopic beats over 48-h Holter recordings.

Variable	Ventricular ectopy		Supraventricular ectopy	
	p-value	95% CI	p-value	95% CI
Phenotype	0.640	− 57.5–90.7	0.162	− 20.7–113.1
Sex	0.540	− 42.3–77.6	0.462	− 35.0–73.2
Age (years)	0.211	− 7.45–31.0	0.634	− 13.4–21.3
Mutant allele count (0–2)	0.026	− 89.1–− 6.60	0.033	− 78.4–− 3.90
NTproBNP (pmol/L)	0.110	− 0.15–1.30	0.223	− 0.26–1.04
Maximal LV wall thickness (mm)	0.130	− 8.14–57.3	0.516	− 20.3–38.7
Presence of LVOTO	0.103	− 157.0–15.9	0.133	− 136.1–19.8

As SDNN is generally considered the Gold Standard measurement of HRV for clinical purposes and cardiac risk stratification^36^, only results for SDNN are presented here. Results for the other time-domain measurements of HRV can found in the Supplementary Material. Cats within the HCM group showed greater HRV compared to cats within the control group, with a higher median SDNN value (22 [17–27] ms vs. 21 [14–26] ms; p = 0.0006). Similarly, there was a significant difference between cats based on mutant allele count, with those cats that had zero mutant alleles for the A31P mutation having a lower median (IQR) SDNN measurement (0 alleles = 20 [14–27] ms, 1 allele = 23 [19–27] ms, 2 alleles=22 [16–28] ms; p = 0.0001). There was no significant difference in SDNN between cats with or without left ventricular outflow tract obstruction (22 [16–26] ms and 22 [16–27] ms; p = 0.29). In the linear mixed model, significant differences in SDNN were also found between cats in the HCM versus control group (p = 0.002) and mutant allele counts (p = 0.0001).

**Figure 3:**
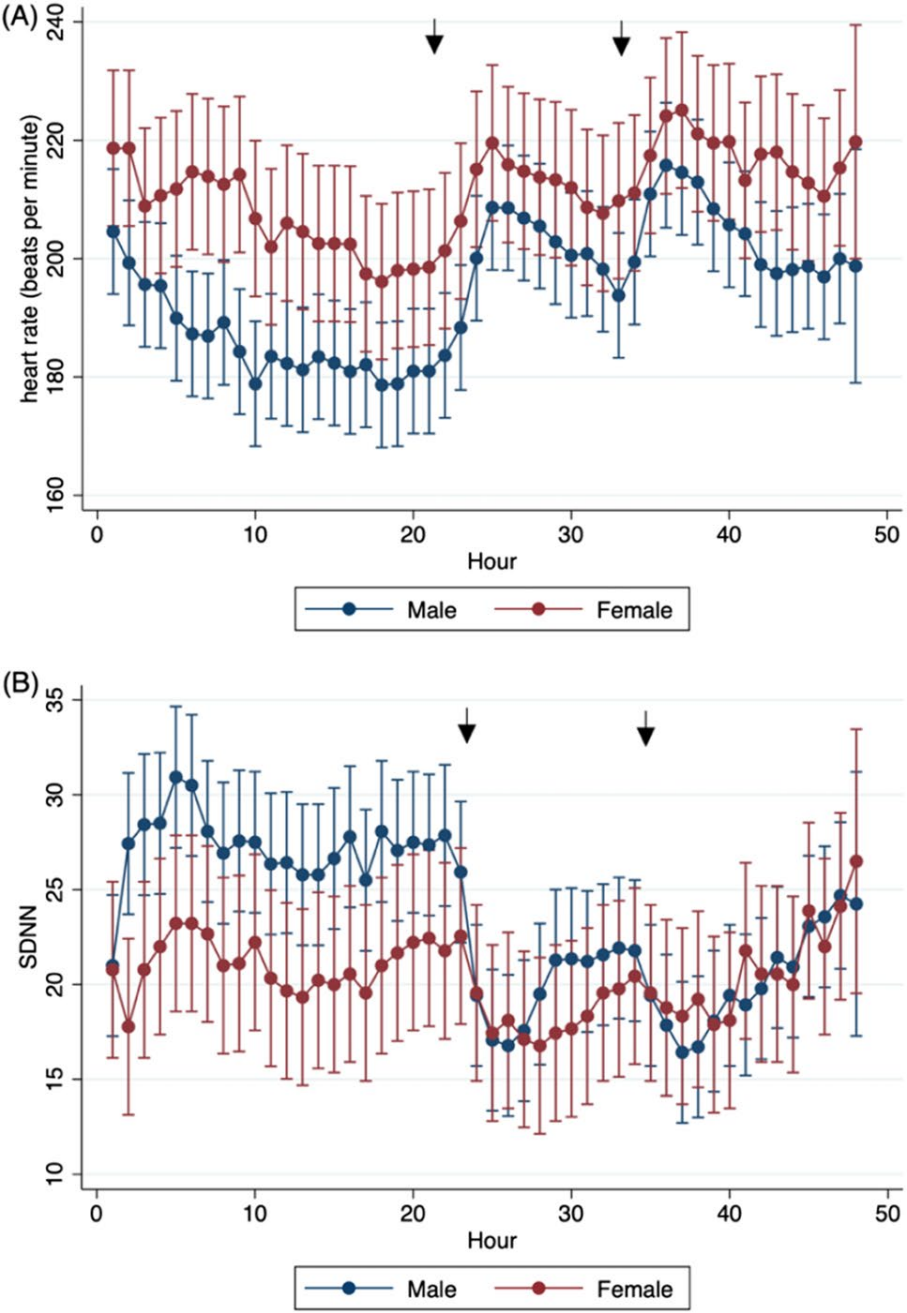
Heart rate and heart rate variability shown between male and female cats. (**A**) Mean heart rate (HR) plotted by hour for the entire AECG recordings separated by sex. (**B**) Standard deviation of NN intervals (SDNN) plotted by hour for the entire AECG recordings separated by sex. Arrows indicate when terbutaline was administered.

Male cats were found to have significantly lower minimum (164 [151–179] vs 172 [155–197] bpm; p = 0.0001), mean (197 [184–212] vs 212 [194–230] bpm; p = 0.0001), and maximum (245 [230–261] vs 259 [242–273] bpm; p = 0.0001) heart rates compared to female cats across the entire AECG recordings (Fig. 3A). While there was a higher percentage of male cats within the HCM group (69%) versus the control group (43%), the difference was not statistically significant (p = 0.36). There was also a significant difference in HRV among sexes, with male cats having a higher median SDNN compared to female cats (22 [18–28] ms vs. 20 [14–25] ms; p = 0.0001). Figure 3B shows the median SDNN for the entire AECG recordings for males and females and demonstrates the higher HRV of males. Figure 3 also demonstrates the effects of terbutaline administration. Namely, in the stressed state (post-terbutaline) mean heart rate for both sexes increased, HRV measured by SDNN decreased and the difference seen between sexes for both mean heart rate and HRV is diminished. Heart rate variability measured by SDNN was significantly lower in the stressed state compared to the resting state (p = 0.0008). Linear mixed model analysis also revealed a significant difference of SDNN between male and female over time before and after terbutaline administration (p = 0.0001) (Fig. 3B).

## Discussion

Our study is the first to design, implement, and validate a pharmacological cardiac stress test in cats with subclinical HCM. Herein, we show that administration of oral terbutaline is feasible and resulted in expected cardiac changes indicative of increased sympathetic drive, presumably what would be seen with physical activity. Namely, mean heart rate significantly increased and HRV significantly decreased. Exercise testing is performed in people with asymptomatic HCM to better assess hemodynamic stability and prognosticate. Furthermore, evaluation of exercise-induced arrhythmias has been shown to provide important information regarding the likelihood of future SCD and survival times^23^. In feline veterinary medicine, an exercise test is not a feasible diagnostic, as our patient population would not be compliant. Therefore, our study aimed to develop a pharmacologic analogue to an exercise test with the goal of inducing increased sympathetic tone.

Despite effectively placing the studied cats in a state of cardiac stress, no effect on supraventricular or ventricular arrhythmia frequency or complexity was seen. This may be because the overall frequency and complexity of arrythmias in the studied population was relatively low or because the cats studied were comprised of relatively mildly affected cats (ACVIM Stage B1 HCM) and healthy controls. The low frequency and complexity of arrhythmias in the studied population is further highlighted when comparing these results to a recent study assessing cats with other non-HCM cardiomyopathies. This study assessed cats with more advanced disease, many in congestive heart failure, and found the median number of ventricular ectopic beats to be 2031 over 24 h^38^. Furthermore, it is possible that no change was seen because the studied population was made up of cats not prone to develop arrhythmias in a stressed state. A larger, longitudinal study that includes cats with both subclinical and later stage HCM would be helpful in determining if our pharmacologic stress test is able to reveal cats that are more prone to arrhythmogenesis and at a greater risk of SCD. Additionally, 24 h of AECG monitoring may not have been sufficiently long enough to evaluate arrhythmogenesis in our study population, especially given day-to-day variability in arrhythmia frequency, and future studies should consider longer monitoring intervals.

Two prior studies have compared the arrhythmia frequency and severity of cats with asymptomatic HCM to healthy cats using 24-h AECG monitoring^20,21^. While one of these studies found that cats with asymptomatic HCM had more frequent and complex ventricular and supraventricular arrhythmias, the other failed to find a significant difference between groups^20,21^. We hypothesized that a possible explanation for these contradictory results was that the first study noted poor AECG device tolerance and therefore, that cats with asymptomatic HCM may be more susceptible to the development of arrhythmias when in a stressed state. However, our results do not support this hypothesis, as subclinical HCM cats did not become significantly more arrhythmic following administration of terbutaline. More recently, 24-h AECG monitoring was used to compare arrhythmia frequency and complexity between cats with decompensated HCM, compensated HCM, and healthy control cats^22^. This study found that cats with both decompensated and compensated HCM had significantly more ventricular ectopy compared to healthy cats but did not find a significant difference between the two HCM groups or a correlation between ectopy and prognosis over the one-year follow-up period. However, of the four cats in this study that died suddenly, three of them had complex arrhythmias: two with atrial fibrillation and ventricular ectopy and one with ventricular tachycardia. As our study was not longitudinal in design, we cannot infer a relationship between arrhythmia development and the likelihood of SCD. However, this study is the first to show a correlation between the frequency of ventricular ectopy on AECG monitoring in cats and MYBPC3 mutant allele count positivity. While statistically significant, this finding should be further evaluated as the low overall frequency of ventricular ectopy in our results and the known day-to-day variability of arrhythmia frequency on 24-h AECG in other species could have resulted in type I error. Further prospective, longitudinal studies are needed to evaluate the relationship between genotype, phenotype, arrhythmogenesis and clinical outcome.

To our knowledge, HRV has not been previously studied in cats with HCM. One study previously assessed HRV in healthy cats comparing spectral HRV parameters in the hospital versus in home settings^33^. Interestingly, our results found that cats with an HCM phenotype had greater HRV and lower mean heart rates compared to healthy controls. Additionally, all of the cats with a sinus arrhythmia present on AECG had an HCM phenotype, which would suggest a relatively higher level of vagal tone. These findings contrast with what was expected given previous studies of HRV in humans with cardiac disease. For example, SDNN was found to be reduced in patients with left ventricular hypertrophy secondary to aortic valve stenosis, HCM or hypertension and the extent of hypertrophy was a positive predictor for the degree of depression of HRV^39^. However, our findings may be resultant of the mild degree of disease in the studied population. Additional studies in people have shown that reduced HRV is a predictor of death for those patients hospitalized for congestive heart failure and of left ventricular dilation in patients suffering myocardial infarction. These patient populations represent more severe categories of disease with clinical signs and hemodynamic compromise. Therefore, our results are likely representative of a compensated population of cats with mild subclinical HCM compared to healthy controls. We hypothesize that with disease progression to a clinical state, HRV would decrease as vagal influence is overridden by an increase in sympathetic tone. Again, prospective studies that encompass both subclinical and decompensated cats with HCM are needed to assess this. If this were the case, then repeated monitoring for decreases in HRV could provide indication of worsening disease even before a clinical state. Furthermore, mean SDNN measurements for all cats in this study were lower than expected for people, whom should have a SDNN greater than 50 ms in health^36^. We propose that this is due to higher mean heart rates in cats. A previous study assessing heart rates and HRV in rhesus macaques found mean SDNN measurements of 27.3 ms for the monkeys with left ventricular hypertrophy and 35.0 ms for the healthy control group^35^. As the mean heart rates of the monkeys were between 136 and 147 bpm, which more closely reflects a cat’s heart rate compared to people, it is plausible that healthy cats have an SDNN below 50 ms. Further studies with larger control groups of healthy cats are needed to assess this.

Male cats had both significantly lower heart rates (minimum, mean, and maximum) and significantly higher HRV compared to female cats. These findings are logical, as individuals with higher mean heart rates are likely to have lower HRV as a results of smaller inter-beat intervals. A previous study assessing heart rates in healthy cats using AECG yielded similar results with females having significantly higher minimum and mean heart rates^40^. These findings are also in alignment with the literature on HRV in people. A large meta-analysis assessing the differences in HRV based on sex in healthy humans found that females had a significantly smaller beat to beat interval, and thus higher heart rate, and lower HRV measured by SDNN compared to males, similar to our findings in this feline population^41^. Furthermore, their findings indicated that although females had higher mean heart rates, they showed greater vagal activity when power spectral density analysis of HRV was performed. It is possible that this higher underlying parasympathetic tone is cardioprotective, despite higher heart rates and lower HRV, as there is a male predisposition in both human and feline HCM. Proposed explanations for this difference in cardiovascular autonomic balance between sexes include differing estrogen levels, differences in oxytocin concentrations, and differences in neural control, specifically via the amygdala and hippocampus. Similar to these human findings, our study highlights sex differences in heart rate, HRV, and cardiovascular autonomic balance in cats. Future studies assessing HRV in cats should ensure to account for these differences between males and females and is a further reminder on the importance of sex balance in study groups.

There were several limitations of this study, largely due to the population of cats included. Firstly, this study included a relatively small number of cats which may result in Type II error. This is particularly possible for the control group, which contained the smallest number of animals. Cats were classified into the control group if they lacked left ventricular hypertrophy on echocardiography and had an NTproBNP < 99 pmol/L. Two cats in this group were heterozygous for the A31P MYBPC3 mutation, and thus it is possible that these cats develop HCM later in life or had histopathologic disease already present that was not appreciable on echocardiography or resulted in an elevated NTproBNP. Additionally, all cats were selected from a closed colony of research animals and therefore results may not be representative of a more general feline population. As such, influences of environment or lifestyle may be different when comparing these animals to typical pet cats with or without HCM. Cats were also sedated for placement of the AECG devices and this may have influenced study results, however the sedation protocol used is relatively short-acting and unlikely to have significantly impacted results beyond the first hour^42^. Furthermore, cats were not acclimated to the AECG devices and had never experienced the placement of one prior to this study. The mean heart rates, both in the resting and stressed state, are higher in our study than those found in previous studies evaluating both client-owned, healthy cats in the home environment and cats with HCM in the home environment^20,33,40^. Despite attempts to minimize handling, use sedation, and place cats back into an enclosure in the same building for the AECG recording, this may reflect a component of environmental stress from the colony environment, placement of the AECG device, and/or administration of oral terbutaline which could confound our results. Finally, there were limitations pertaining to the equipment and software used to obtain and analyze the AECG recordings. In particular, the software used did not provide options to perform spectral analysis of HRV and therefore this limits our ability to draw conclusions about sympathetic and vagal tone in the study population or compare our results to the previously published literature on spectral HRV in healthy cats.

In the present study, we show that administration of oral terbutaline is an effective and feasible cardiac stress test in cats with subclinical HCM. Following dosage with oral terbutaline, heart rates increased and HRV decreased indicating a predictable pattern of increased sympathetic tone. A low number of single ventricular premature complexes was relatively common in the studied population of cats, however few cats had more frequent or complex arrhythmias. Arrhythmias also did not differ before or after terbutaline. Cats with an HCM phenotype had lower heart rates and higher HRV compared to control cats. Furthermore, we found significant differences in heart rate and HRV between male and female cats, with male cats having lower heart rates and higher HRV. Finally, cats positive for the A31P MYBPC3 mutation were more likely to have ventricular ectopy. This finding may indicate that client-owned cats known to be heterozygous or homozygous for the A31P mutation should be more proactively screened for arrhythmias compared to animals that are wild type. Further studies that include more severe manifestations of HCM and a larger control group are necessary to determine if such clinical recommendations are warranted.

## Supplementary Information

Supplementary Table 1.
