# Supplementary material for: Ambulatory electrocardiography, heart rate variability, and pharmacologic stress testing in cats with subclinical hypertrophic cardiomyopathy
Source: Sci Rep. 2022 Feb 4;12:1963. doi: 10.1038/s41598-022-05999-x (PMC8817045; doi:10.1038/s41598-022-05999-x)
Supplement: Supplementary file 1 — Supplementary Table 1. [file 41598_2022_5999_MOESM1_ESM.pdf]

Supplementary Material

Phenotype	minimum HR	mean HR	maximal HR	pNN50	RMSSD	SDNN	SDANN	Tri
**Control**	169 (156 - 187)	201 (186 - 218)	252 (236 - 266)	1 (0 - 6)	17.5 (11 - 27)	21 (14 - 26)	10 (6 - 15)	6 (4 - 8)
**HCM**	165 (150 - 181)	202 (186 - 217)	250 (233 - 266)	2 (0 - 5)	20 (15 - 27)	22 (17 - 27)	12 (8 - 17)	6 (4 - 7)
**P-value**	0.0007	0.72	0.98	0.041	0.0002	0.0006	0.0001	0.075
**Obstruction**	**minimum HR**	**mean HR**	**maximal HR**	**pNN50**	**RMSSD**	**SDNN**	**SDANN**	**Tri**
**No LVOTO**	166 (153 - 181)	201 (186 - 216)	251 (235 - 266)	1 (0 - 4)	18 (13 - 24)	22 (16 - 27)	12 (8 - 17)	6 (4 - 8)
**LVOTO**	166 (151 - 193)	202 (185 - 225)	250 (232 - 266)	3 (0 - 10)	22 (15 - 32)	22 (16 - 26)	11 (6 - 16)	5 (4 - 7)
**P-value**	0.055	0.022	0.26	0.0001	0.0001	0.29	0.017	0.0015
**Sex**	**minimum HR**	**mean HR**	**maximal HR**	**pNN50**	**RMSSD**	**SDNN**	**SDANN**	**Tri**
**Male**	164 (151 - 179	197 (184 - 212)	245 (230 - 261)	2 (0 - 6)	20 (15 - 28.5)	22 (18 -28)	12 (8 - 17)	6 (5 - 8)
**Female**	172 (155 - 197)	212 (194 - 230)	259 (242 - 273)	1 (0 - 5)	16 (12 - 25)	20 (14 - 25)	10 (6 -16)	5 (4 - 7)
**P-value**	0.0001	0.0001	0.0001	0.0001	0.0001	0.0001	0.0001	0.0001
**Genotype**	**minimum HR**	**mean HR**	**maximal HR**	**pNN50**	**RMSSD**	**SDNN**	**SDANN**	**Tri**
**WT**	170 (155 - 187)	201 (186 - 218)	247 (232 - 261)	1 (0 -7)	18 (12 -29)	20 (14 - 27)	10 (7 - 15)	5 (4 - 8)
**Het**	165 (155 - 177)	204 (194 - 216)	260 (247 - 272)	1 (0 - 3)	18 (14 - 22)	23 (19 - 27)	13 (10 - 18)	6 (5 - 8)
**Ho**	161 (144 - 188)	198 (179 - 222)	227 (244 - 263)	3 (1 - 9)	22 (15 - 32)	22 (16 - 28)	11 (7 - 16)	5 (4 - 7)
**P-value**	0.0002	0.0053	0.0001	0.0001	0.0001	0.0001	0.0001	0.0001

Supplementary Table 1. Displayed are the statistical results of heart rate and all the heart rate variability parameters evaluated. Analyses were conducted for phenotype, presence or absence of left ventricular outflow tract obstruction, sex, and genotype.
